# Influence of Obesity and IL-6 on Human Postprandial Amino Acid and Protein Metabolism at Whole-body and Tissue Level

**DOI:** 10.1210/clinem/dgaf547

**Published:** 2025-10-09

**Authors:** Beckey Trinh, Signe Johanne Rasmussen, Mathilde Ehnhuus Brøgger-Jensen, Ana Rita Albuquerque de Almeida Tavanez, Anton Lund, Alexandra Vassilieva, Susanne Janum, Ulrik Winning Iepsen, Kirsten Møller, Bente Klarlund Pedersen, Gerrit Van Hall, Helga Ellingsgaard

**Affiliations:** The Centre for Physical Activity Research, Rigshospitalet, University of Copenhagen, 2100 Copenhagen, Denmark; The Centre for Physical Activity Research, Rigshospitalet, University of Copenhagen, 2100 Copenhagen, Denmark; The Centre for Physical Activity Research, Rigshospitalet, University of Copenhagen, 2100 Copenhagen, Denmark; The Centre for Physical Activity Research, Rigshospitalet, University of Copenhagen, 2100 Copenhagen, Denmark; Department of Neuroanaesthesiology, Rigshospitalet, University of Copenhagen, 2100 Copenhagen, Denmark; Department of Neuroanaesthesiology, Rigshospitalet, University of Copenhagen, 2100 Copenhagen, Denmark; Department of Neuroanaesthesiology, Rigshospitalet, University of Copenhagen, 2100 Copenhagen, Denmark; The Centre for Physical Activity Research, Rigshospitalet, University of Copenhagen, 2100 Copenhagen, Denmark; Department of Anaesthesia and Intensive Care, Copenhagen University Hospital—Hvidovre, 2650 Hvidovre, Denmark; Department of Neuroanaesthesiology, Rigshospitalet, University of Copenhagen, 2100 Copenhagen, Denmark; The Neuroscience Center and Institute for Clinical Medicine, Rigshospitalet, University of Copenhagen, 2100 Copenhagen, Denmark; The Centre for Physical Activity Research, Rigshospitalet, University of Copenhagen, 2100 Copenhagen, Denmark; Department of Clinical Biochemistry, Rigshospitalet, University of Copenhagen, 2100 Copenhagen, Denmark; Clinical Metabolomics Core Facility, Rigshospitalet, 2100 Copenhagen, Denmark; Department of Biomedical Sciences, University of Copenhagen, 2100 Copenhagen, Denmark; The Centre for Physical Activity Research, Rigshospitalet, University of Copenhagen, 2100 Copenhagen, Denmark

**Keywords:** protein turnover, stable isotopes, skeletal muscle, adipose tissue, tocilizumab, meal

## Abstract

**Context:**

Sarcopenic obesity, the loss of muscle mass and function in people with obesity, may result from altered muscle protein synthesis and degradation. Chronic low-grade inflammation, particularly IL-6, has been implicated.

**Objective:**

To assess the role of IL-6 in protein and amino acid metabolism during fasting and postprandial states in humans with healthy weight or obesity at whole-body, skeletal muscle, and subcutaneous adipose tissue levels.

**Methods:**

In this placebo-controlled, nonrandomized, participant-blinded study, 12 men with healthy weight and 12 men with obesity received placebo (0.9% saline) or 3 weeks of IL-6 receptor blockade with tocilizumab. Isotope dilution/incorporation techniques and arteriovenous balance measurements were applied in fasted and postprandial states. The trial was originally designed to examine IL-6 effects on fat storage (reported previously). Here, we present prespecified exploratory outcomes on amino acid and protein turnover.

**Results:**

Obesity was associated with reduced meal-induced muscle-protein gain driven by impaired suppression of muscle protein degradation, and with reduced appearance of amino acids from meals. In both groups, IL-6 receptor blockade increased fasting and postprandial plasma amino acids and reduced postprandial plasma protein synthesis without affecting skeletal muscle protein turnover. In the healthy weight group, it also increased amino acid appearance from the meal and postprandial phenylalanine oxidation.

**Conclusion:**

Obesity impairs meal-induced muscle-protein gain, through insufficient suppression of protein degradation. Basal IL-6 activity does not regulate muscle protein turnover but influences amino acid metabolism and protein synthesis in extramuscular tissues.

Sarcopenic obesity, defined as the loss of muscle mass and function in people with obesity, is a complex and poorly understood condition (1, 2). Alterations in muscle protein synthesis, degradation, or both, are thought to contribute to its development, but the underlying drivers remain unclear. Chronic low-grade inflammation, common in obesity, may disrupt protein turnover and contribute to sarcopenia (3).

Among the cytokines elevated in obesity, IL-6 is of particular interest, with circulating concentrations typically <10 pg/mL (4). Evidence from cell culture and animal studies, mainly in cancer and sepsis models (3, 5-7), suggests that IL-6 may promote muscle wasting. In humans, catabolic effects has primarily been demonstrated in a study where recombinant human IL-6 infusion raised plasma IL-6 levels to ∼140 pg/mL (8) and decreased muscle protein turnover, which was likely secondary to reduced plasma amino acid availability rather than a direct effect on muscle. In rheumatoid arthritis, where IL-6 levels are elevated, IL-6 receptor (IL-6R) blockade increases lean mass (9, 10). However, IL-6 concentrations in rheumatoid arthritis (11, 12) and cancer (13) are substantially higher than in obesity, limiting direct extrapolation.

Conversely, under healthy conditions, IL-6 appears necessary for muscle hypertrophy and satellite cell proliferation, as shown in mice (14) and human cell cultures (15), indicating a context-dependent role for IL-6 in muscle physiology. Recently, we showed that 3 weeks of IL-6R blockade with tocilizumab in men with healthy weight or obesity did not alter whole-body protein turnover (16) but increased plasma amino acid concentrations (16), suggesting that even low basal IL-6 (plasma concentrations < 1 pg/mL) may influence amino acid metabolism and potentially muscle mass maintenance in humans.

Feeding, particularly amino acid intake combined with insulin, strongly stimulates muscle protein gain by enhancing synthesis and reducing degradation (17). Most studies report similar or lower muscle protein synthesis rates in response to protein-rich meals or amino acid infusions in obesity compared to healthy weight (18). Protein degradation has been examined in only 1 study, which found more effective suppression across the leg in obesity during insulin and amino acid infusion (19), suggesting lower protein turnover. The role of basal IL-6 activity in postprandial amino acid and protein metabolism remains unexplored.

This study was originally designed to assess IL-6 effects on fat storage during fasting and postprandial states (20). Here, we report prespecified exploratory outcomes examining IL-6's role in amino acid and protein metabolism at whole-body, skeletal muscle, and subcutaneous adipose tissue level in men with healthy weight or obesity using isotope dilution/incorporation techniques and arteriovenous balance measurements.

We hypothesized that the obesity-related basal increase in IL-6 impairs net muscle protein gain in individuals with obesity, and that IL-6R blockade would therefore improve net muscle protein gain in this group only. Given the multiple pathways influencing net muscle protein gain, we conducted exploratory, hypothesis-generating analyses to assess the effect of IL-6R blockade across a broad set of amino acid and protein kinetic parameters. Because amino acid intake is a strong stimulator of muscle protein gain, we studied these parameters in a meal context.

## Methods

### Study Design

This was a placebo-controlled, nonrandomized, participant-blinded study. The primary aim of this study, which is covered elsewhere (20), was to investigate the role of IL-6 in regulating lipid and glucose kinetics before and after a standard liquid meal in men with either normal weight or obesity. A separate distinctive aim was to study amino acid and protein turnover with prespecified exploratory analyses, the results of which are reported here.

Inclusion criteria were male gender, age ≥ 18 and ≤ 40 years, stable weight in the past 6 months, and body mass index (BMI) ≥ 18 and < 25 kg/m^2^ for the group with healthy weight and a BMI ≥ 30 and ≤ 40 kg/m^2^ for the group with obesity. Participants were healthy based on medical history and clinical and basic chemistry assessment, not on therapy with corticosteroids, and not using nonsteroidal anti-inflammatory drugs or paracetamol. The groups were matched for age. On the screening day, an electrocardiogram, measurements of blood pressure, height, weight, hip, waist, body composition (dual X-ray absorptiometry, Lunar Prodigy; GE Medical Systems, Madison, WI, USA), cardiorespiratory fitness (VO_2peak_ test), and oral glucose tolerance (with 75 g of glucose) as well as baseline blood samples were obtained.

**Figure 1.**
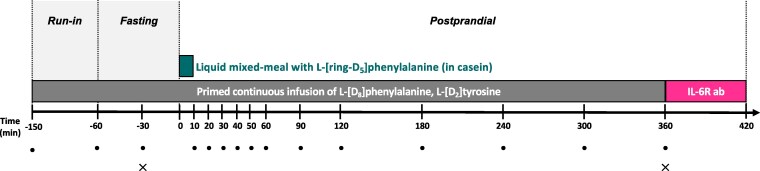
Overview of study visit. At visit Saline, the IL-6 receptor antibody tocilizumab was infused from 360 minutes to 420 minutes. IL-6R ab, IL-6 receptor antibody; • blood sample; × skeletal muscle biopsy.

Participants were studied on 2 separate occasions 21 days apart. An overview of the procedures at the 2 study visits is given in Fig. 1.

On both study visits, participants presented fasted (≥10 hours, no alcohol or caffeine) at 7:00 Am. After voiding, body weight was measured and participants were placed in bed in a temperature-regulated room for the entire study visit (20-24 °C, mean difference between study visits within-individual 0.5 °C). Participants were permitted to drink water ad libitum during the experiments.

Each study visit consisted of 3 phases: a run-in phase (-150 to -60 minutes), during which isotopic equilibrium was achieved, a fasting phase (-60 to 0 minutes) and a postprandial phase (0 to 360 minutes). An antecubital venous catheter was placed in the right arm, from which the first baseline blood sample (at -150 minutes) was obtained. Thereafter, the venous catheter was used for infusion of stable isotope-labeled tracers. A primed, continuous infusion of tracers was started at -150 minutes. Then, a catheter was placed in the left radial artery, a second catheter was placed in the right common femoral vein, and a third catheter was placed in a superficial epigastric vein for collection of sequential blood samples. A liquid mixed meal was ingested at 0 minutes. Biopsies from the right vastus lateralis muscle were obtained at -30 and 360 minutes.

Saline 0.9% served as placebo on the first study visit (Saline), and the IL-6 receptor antibody, tocilizumab, was used to block IL-6 activity on the second study visit (IL-6R ab). The order of the 2 study visits was not randomized because of the long elimination half-life of tocilizumab (t_½_ after first dose = 123 ± 41.5 hours (21)) and therefore only the participants were blinded. Tocilizumab was infused at the end of visit Saline, which allowed us to study the chronic (ie, 3-week) effects of IL-6 receptor blockade on visit IL-6R ab. The 3-week timeframe was chosen because we have previously observed increased plasma amino acid concentrations already after 3 weeks of IL-6 receptor blockade (16) and therefore anticipated to find altered amino acid or protein metabolism at this point.

The study was approved by the Ethics Committee of Copenhagen and Frederiksberg Communities, Denmark, reported to the Danish Data Protection (P-2020-1209), registered at Clinicaltrials.gov (NCT04687540), and performed according to the Declaration of Helsinki. All participants gave written informed consent.

### Modeling Insulin Sensitivity and Secretion

Baseline fasting insulin (pmol/L) and glucose concentrations (mmol/L) were taken from blood samples at -150 minutes on visit Saline as data sets were more complete than from samples at screening.

Insulin concentrations (pmol/L) and glucose concentrations (mmol/L) after ingestion of the liquid mixed-meal at each study visit (at 0-120 minutes) were used for the assessment of insulin sensitivity and secretion.

Whole-body insulin sensitivity (Matsuda index) was calculated as follows (22):


$$Matsuda\;index=\frac{10\;000}{\sqrt{\frac{insulin_{mean}\times glucose_{mean}\times \;18}{7.18}\times \frac{insulin_{\;fasting}\times glucose_{\;fasting}\times 18}{7.18}}}$$

Insulin resistance was assessed with homeostasis model assessment-insulin resistance (HOMA-IR) index (23)


$$HOMA-IR=\;\frac{glucose_{\;fasting}\times insulin_{\;fasting}}{22.5\times 7.18}$$

### Diet and Physical Activity

Participants were instructed to maintain their free-living physical activity and eating habits during the 3-week study period. To control food intake, participants were encouraged to use self-reported dietary records for 48 hours before visit Saline and to consume a similar diet before visit IL-6R ab.

### Tocilizumab

Tocilizumab (RRID:AB_2459656, Roche, Basel, Switzerland), a recombinant humanized monoclonal IL-6 receptor antibody, was reconstituted in 100 mL saline 0.9% and infused over 60 minutes at a dose of 8 mg/kg body weight or a maximum of 800 mg.

### Catheters

An arterial catheter (BD Arterial Cannula with FloSwitch, 20G 1.1 × 45 mm) was inserted in the left radial artery under sterile conditions, local anesthesia with 0.5 to 1 mL lidocaine (20 mg/mL), and ultrasound guidance. A central venous catheter (Teleflex Arrow One-Lumen CVC set, 20 GA × 12 cm) was inserted retrogradely into the right common femoral vein under local anesthesia with 5 mL lidocaine (20 mg/mL) and ultrasound guidance. Both catheters were kept patent with normal saline using a pressure system (Salter Labs InfuseIT pressure infusor 500 mL, IL, USA). A superficial epigastric vein was catheterized with a 20G peripheral venous catheter (32 mm or 64 mm, depending on individual anatomy) under ultrasound guidance, threaded toward the groin with the tip located proximal to the inguinal ligament. The catheter was kept patent by continuous infusion of normal saline at a rate of 40 mL/h.

### Blood Flow

Right femoral arterial blood flow was measured simultaneously to each blood sample (except at -150 minutes) using ultrasound Doppler (Vivid E9, GE Healthcare, Pittsburgh, PA) equipped with a linear probe (L9) operating at an imaging frequency of 4/8 MHz and a Doppler frequency at 4.2 MHz, as described previously (24, 25). The site of measurement at the common femoral artery was distal to the inguinal ligament but above the bifurcation to avoid turbulence at the bifurcation. Mean blood flow velocities were measured continuously in duplex mode and averaged over ∼30 seconds at the lowest possible insonation angle (<60°). Sample volume was maximized to the width of the vessel. A low-velocity filter (<1.8 m/s) rejected noise caused by turbulence at the vascular wall. For each Doppler recording, arterial diameter was measured with the probe parallel to the vessel during systole from B-mode images.

### Blood Samples

At each study visit, a baseline blood sample was obtained from the antecubital venous catheter at -150 minutes. All subsequent blood samples for the calculation of substrate kinetics were collected simultaneously from arterial, femoral, and epigastric catheters at -60, -30, 10, 20, 30, 40, 50, 60 90, 120, 180, 240, 300, and 360 minutes. Blood samples for hormones and cytokines were obtained from the arterial catheter at -30, 20, 30, 60 90, 120, 180, 240, 300, and 360 minutes. All blood samples were collected in ice cold EDTA tubes and immediately centrifuged (1.9*g*, 10 minutes, 4 °C) to obtain plasma. Plasma was left on dry ice until stored at -80 °C.

### Biopsies

Skeletal muscle biopsies from the right vastus lateralis muscle were obtained under sterile conditions and local anesthesia (2-5 mL lidocaine 20 mg/mL) using the Bard MAGNUM Biopsy Instrument (12 GA, 10 cm, C. R. Bard, Inc., AZ, USA). Biopsies were immediately snap-frozen in liquid nitrogen and kept at -80 °C until further analysis. Before analysis, muscle biopsies were freeze-dried during at least 48 hours and dissected from apparent nonmuscle tissue under a microscope.

### Sample Analyses

Stable isotope-labeled tracers in plasma and muscle biopsies were quantified at the Clinical Metabolomics Core Facility at Rigshospitalet, as previously described (26-28).

Insulin, GH, and cortisol concentrations were measured at the Department of Clinical Biochemistry at Rigshospitalet. Insulin, c-peptide, GH, and cortisol were analyzed using the COBAS 8000 e801 immunoassay system (Roche Diagnostics GmbH); paracetamol was analyzed with the COBAS 8000 c502 photometric assay system. Glucagon was measured by ELISA (RRID:AB_2737304, Mercodia, Uppsala, Sweden). IL-6 and TNF-α were measured at -150, -30, 20, 30, 60 90, 120, 180, 240, 300, and 360 minutes on both study visits by multiarray electrochemiluminescence (V-PLEX, Meso Scale Diagnostics, Rockville, MD, USA).

### Stable Isotopes and Substrate Kinetics

All stable isotope-labeled tracers were purchased from Cambridge Isotope Laboratories (Andover, MA, USA). After obtaining baseline blood samples for the measurement of background enrichment, a primed-continuous infusion of L-[D_8_]phenylalanine (prime 3 μmol/kg lean mass [LM], continuous 0.07 μmol/kg LM/min), and L-[ring-3,5-D_2_]tyrosine (prime 2.3 μmol/kg LM, continuous 0.04 μmol/kg LM/min) were started and maintained for 360 minutes.

The liquid mixed meal (400 mL, 631 kcal, carbohydrate 48 E%, protein 17 E%, and fat 35 E%) was composed of glucose (80 g dextrose + 2.5 g [U-^13^C_6_]glucose), rapeseed oil (25 g), K-[U-^13^C_16_]palmitate (0.2 g), and casein intrinsically labeled (26 g) with L-[ring-D_5_]phenylalanine and [5,5,5-D_3_]leucine. Intrinsically labeled casein is produced via the infusion of L-[ring-D_5_]phenylalanine and [5,5,5-D_3_]leucine into lactating Holstein dairy cows to obtain L-[ring-D_5_]phenylalanine- and [5,5,5-D_3_]leucine-enriched milk, from which the casein fraction is obtained at Arla Foods (Nørre Vium, Denmark) following a previously described procedure (29). The meal was consumed within 5 minutes under supervision.

#### Calculations

For each participant and study day, the actual isotope infusion rate was calculated from the infusate concentration multiplied by the infusion flow rate.

*Protein kinetics.* All kinetics parameters were normalized to LM determined by dual x-ray absorptiometry.

For phenylalanine enrichment, L-[D_8_]phenylalanine and its transamination product L-[D_7_]phenylalanine and its oxidation products (L-[D_7_]tyrosine and L-[D_6_]tyrosine) were measured. The sums of D_8_- and D_7_-phenylalanine as well as D_7_- and D_6_-tyrosine were used for all calculations.

Whole-body *R_a_* and *R_d_* of total phenylalanine and tyrosine were calculated using the non-steady-state equations of Steele (30) adapted for stable isotopes (31): t


$$R_{a\;total}=\frac{F-pV\left(\frac{C_{2}+\;C_{1}}{2}\right)\left(\frac{E_{2}-\;E_{1}}{t_{2}-\;t_{1}}\right)}{\frac{E_{2}+\;E_{1}}{2}}$$

$$R_{d\;total}=R_{a\;total}-pV\left(\frac{C_{2}-\;C_{1}}{t_{2}-\;t_{1}}\right)$$

where *F* is the tracer infusion rate (µmol/min), *E_1_* and *E_2_* are the blood tracer enrichment of L-[D_8_] plus [D_7_]phenylalanine or L-[ring-3,5-D_2_]tyrosine (TTR), at time 1 (*t_1_*) and 2 (*t_2_*) (min), respectively, and *C_1_* and *C_2_* are the plasma concentrations (µmol/L) at *t_1_* and *t_2_* (min), respectively. *pV* is the volume of distribution, 0.125 (kg body weight)^-1^ for phenylalanine and tyrosine.

Since the liquid mixed meal contained casein labeled with L-[ring-D_5_]phenylalanine, the appearance of meal-derived amino acids in circulation can be estimated by measuring the appearance of L-[ring-D_5_]phenylalanine in circulation. *R_a_* of oral phenylalanine was calculated as follows:


$$oral\;R_{a}phenylalanine=R_{a\;total}\times \;\frac{E_{2}+\;E_{1}}{2}+\frac{\;pV\left(\frac{E_{2}-\;E_{1}}{t_{2}-\;t_{1}}\right)}{r}\;$$

where *E_1_* and *E_2_* are the blood isotope enrichment (TTR) of L-[ring-D_5_]phenylalanine at time 1 (*t_1_*) and 2 (*t_2_*) (min), respectively, and *r* is the ratio between L-[ring-D_5_]phenylalanine to unlabeled phenylalanine in the meal.

The appearance of oral phenylalanine after splanchnic extraction over 6 hours was calculated as the area under the curve (AUC) of oral *R_a_* phenylalanine in percentage of the amount of ingested phenylalanine.

Phenylalanine is an essential amino acid and protein degradation is the sole source of endogenous phenylalanine released into circulation (32). Thus, *R_a_* of endogenous phenylalanine (µmol/kg LM/min) is representative of protein degradation and is calculated as follows:


$$R_{a\;endo}=F-\;\frac{\;pV\left(\frac{R_{a\;total\;2}+\;R_{a\;total\;1}}{2}\right)\;\times \frac{E_{2}-\;E_{1}}{C_{2}-\;C_{1}}\;}{\frac{E_{2}+\;E_{1}}{2}}\;-\;R_{a\;oral}$$

where *F* is the infusion rate of L-[D_8_]phenylalanine (µmol/min), *E_1_* and *E_2_* are the blood isotope enrichment (TTR) of both L-[D_7_]phenylalanine and L-[D_8_]phenylalanine at time 1 (*t_1_*) and 2 (*t_2_*) (min), and *C_1_* and *C_2_* are the plasma concentrations of endogenous phenylalanine (µmol/L) at *t_1_* and *t_2_* respectively. *pV* is the volume of distribution, 0.125 (kg body weight)^−1^ for phenylalanine.

Phenylalanine is catabolized almost exclusively through hydroxylation to tyrosine (first and rate-limiting step in phenylalanine oxidation), whereas no conversion of tyrosine to phenylalanine is possible (33). The hydroxylation rate (µmol/kg LM/min) can be quantified using the enrichment of D_7_- and D_6_-tyrosine which appear from the hydroxylation of L-[D_8_]phenylalanine and its transamination product L-[D_7_]phenylalanine:


$$Phe\;hydroxylation=R_{a\;D2tyr}\times \;\frac{E_{D7D6tyr}}{\frac{E_{D8D7phe\;2}+\;E_{D8D7phe\;1}}{2}}$$

where *E_D7D6tyr_* is the plasma enrichment (TTR) of both L-[D_7_]tyrosine and L-[D_6_]tyrosine, *E_D8D7phe_* is the plasma enrichment of L-[D_8_]phenylalanine and L-[D_7_]phenylalanine, and *R_a D2tyr_* the *R_a_* of L-[ring-3,5-D_2_]tyrosine (µmol/kg LM/min).

Therefore, assuming that the only pathways of phenylalanine removal (*R_d total_*) are protein synthesis and hydroxylation to tyrosine, the rate of protein synthesis is given by:


$$Protein\;synthesis=R_{d\;total}-Phe\;hydroxylation$$

Total protein degradation and synthesis rates in grams per day is estimated by dividing the previously mentioned rates by the average phenylalanine content in protein (273 μmol/g protein) and multiplying by 60 × 24 minutes. Net protein loss (g/d) equals protein degradation minus protein synthesis.

Muscle and total plasma protein fractional synthesis rate (FSR) was calculated using the standard precursor-product model based on the incorporation of L-[ring-D_5_]phenylalanine, L-[ring-D_7_]phenylalanine, and L-[ring-D_8_]phenylalanine into protein:


$$FSR\;(\text{\%}/h)=\frac{E_{\;protein\;2}-E_{\;protein\;1}}{E_{\;precursor}\;\times \;(t_{2}-\;t_{1})}\times 100\text{\%}$$

where *E_protein 1_* and *E_protein 1_* are L-[ring-D_5_]phenylalanine, L-[D_7_]phenylalanine, and L-[D_8_]phenylalanine enrichment in the myofibrillar proteins at time 1 (*t_1_*) and 2 (*t_2_*) (h) respectively. *E_precursor_* is the weighted average enrichment of L-[ring-D_5_]phenylalanine, L-[D_7_]phenylalanine and L-[D_8_]phenylalanine in the muscle free pool (for muscle protein) or plasma (for plasma protein) during the incorporation time. Muscle protein FSR was measured at time -30 and 360 minutes, plasma protein FSR was measured at times 60, 120, 240, and 360 minutes.

*Leg kinetics*


$$Net\;leg\;balance=(C_{a}-C_{v})\times blood\;flow$$

where *C_a_* is the plasma concentration in the artery, *C_v_* is the plasma concentration (µmol/L) in the femoral vein and *blood flow* is the blood flow in the femoral artery (L/min).

Leg uptake and release were calculated as follows:


$$Fractional\;extraction\;(\text{\%})=\;\frac{C_{a}\times E_{a}-C_{v}\times E_{v}}{C_{a}\times E_{a}}\times 100\text{\%}$$

$$Uptake=Fractional\;extraction\times C_{a}\times blood\;flow$$

Release=uptake−netbalance

where *C_a_* and *C_v_*, *E_a_* and *E_v_* are concentrations (µmol/) and enrichments (TTR) in the radial artery and femoral vein, respectively.

### Sample Size

No study has previously investigated postprandial substrate metabolism in the presence of IL-6R blockade. Therefore, the sample size of this study was determined based on previous studies where stable isotopes have been used to study substrate kinetics in the context of a meal, IL-6 infusion, or IL-6R blockade during exercise (8, 16, 34, 35).

### Statistical Analysis

Data were analyzed in R studio (R version 4.1.2, R studio version 2021.09.1) (36, 37), using the “lme4” (38) and “emmeans” (39) packages. AUC were calculated using the “flux” package (40) after missing values were imputed with the “zoo” package (41), where applicable, and GraphPad Prism (GraphPad Software, Boston, MA, USA; version 10.0.0 for Windows).

Analyses were prespecified exploratory outcomes comprising physiological and biochemical measures intended to be hypothesis-generating. Therefore, unadjusted *P* values, effect sizes, and corresponding 2-sided 95% CIs are provided to aid interpretation. Given the exploratory nature of these analyses, *P* <.05 should not be interpreted as statistically significant in the confirmatory sense, but rather as signals warranting further investigation.

Descriptive statistics are represented as mean ± SD. Baseline characteristics, safety parameters, and baseline values between visits and between groups were compared using paired *t*-tests and Welch 2-sample *t*-test, respectively. For postprandial effects, amino acid concentrations averaged over -60 and -30 minutes, kinetics measurements at -45 minutes, and hormones and IL-6 concentrations at -30 minutes were considered baseline values. We applied repeated-measures linear mixed-effects models, using time, group, time:group interaction as categorical fixed effects and individual participants as a random effect to compare between groups. We applied repeated-measures linear mixed-effects models, using time, IL-6R ab, time: IL-6R ab interaction as categorical fixed effects and individual participants as a random effect to test for IL-6R ab effects, followed by post hoc Dunnett's many-to-one comparisons adjustment to determine differences between each postprandial timepoint to baseline values at visit Saline. Contrasts between visit IL-6R ab and visit Saline at each timepoint were not further adjusted. Normal distribution of the residuals and homogeneity of variances were checked to fulfill the assumptions for using linear models. Variables not meeting model assumptions were logarithmically transformed for optimal model fit. A secondary mixed-effects model using time, IL-6R ab, group, and time × IL-6R ab × group interaction was used to explore differences in effect of IL-6 receptor blockade between the group with healthy weight and the group with obesity. For all outcomes plotted in graphs, the raw mean and standard error of the mean (SEM) are shown for each timepoint. Estimated same-visit and between-visit changes with 95% CI were extracted from the mixed-effects model. For log-transformed variables, differences were analyzed on a log-scale and reported as back-transformed relative difference with 95% CIs. All statistical analyses were done with n = 12 per group.

## Results

### Participants

Between March 16, 2021, and July 29, 2021, 25 male participants were included; 12 participants with healthy weight and 12 participants with obesity completed the study. Details about the recruitment process can be found in the supplemental information (Fig. S1 (42)). Baseline characteristics are given in Table 1. As per study design, the group with obesity had a higher BMI, fat mass (absolute and in percent of soft tissue), waist-to-height ratio, fasting insulin and glucose, insulin resistance (HOMA-IR), and triglycerides but lower high-density lipoprotein cholesterol than the group with healthy weight, HbA1c was similar (Table 1). No participant took any kind of lipid-lowering, glucose-lowering, or anti-inflammatory medication.

**Table 1.** Baseline characteristics

	Healthy weight(n = 12)	Obesity (n = 12)
Age (y)	29.1 ± 5.7	33.8 ± 3.2^*a*^
**Body composition**		
Body weight (kg)	75.4 ± 5.4	111.8 ± 12.1^*a*^
BMI (kg/m^2^)	22.5 ± 1.3	34.6 ± 2.8^*a*^
Hip (cm)	96.3 ± 7.8	116.2 ± 6.0^*a*^
Waist (cm)	81.4 ± 5.6	110.4 ± 7.4^*a*^
Waist-to-height ratio	0.4 ± 0.0	0.6 ± 0.0^*a*^
Total fat mass (kg)	14.0 ± 4.6	39.6 ± 6.9^*a*^
Total fat (% of total soft tissue)	19.3 ± 6.0	36.9 ± 4.6^*a*^
Android fat mass (kg)	1.3 ± 0.4	4.5 ± 1.1^*a*^
Gynoid fat mass (kg)	3 ± 1.1	4.5 ± 1.1^*a*^
Total lean mass (kg)	58.1 ± 5.2	68.0 ± 8.7^*a*^
**Metabolic profile**		
HbA1c (mmol/mol)	33.6 ± 2.1	33.5 ± 2.3
HbA1c (%)	5.2 ± 0.2	5.2 ± 0.2
Fasting glucose (mmol/L)	5.30 ± 0.27	5.67 ± 0.33^*a*^
Fasting insulin (pmol/L)	31.6 ± 9.8^*b*^	89.8 ± 44.9^*a*^
Matsuda Index	5.53 ± 1.63^*b*^	2.78 ± 1.60^*a*^
HOMA-IR	1.29 ± 0.55	3.89 ± 1.61^*a*^
Glucagon (pmol/L)	4.108 ± 1.897	5.950 ± 1.956^*a*^
LDL cholesterol (mmol/L)	2.8 ± 0.7	3.0 ± 0.7
HDL cholesterol (mmol/L)	1.2 ± 0.1	1.0 ± 0.3^*a*^
Triglycerides (mmol/L)	0.87 ± 0.24	1.63 ± 0.82^*a*^
**Cytokines and hormones**		
IL-6 (pg/mL)	0.29 ± 0.12	0.49 ± 0.15^*a*^
TNF-α (pg/mL)	1.43 ± 0.48	1.63 ± 0.53
**Blood pressure**		
Systolic BP (mm Hg)	122.8 ± 6.7	140.7 ± 14.3^*a*^
Diastolic BP (mm Hg)	75.4 ± 4.0	82.2 ± 10.7

Data are presented as mean ± SD. BP, blood pressure; HDL, high-density lipoprotein; LDL, low-density lipoprotein; OGTT, oral glucose tolerance test.

^*a*^ *P* < .05 in Welch 2-sample t-test, *P* values are unadjusted for multiple testing and should be interpreted in the context of exploratory, hypothesis-generating analyses.

^*b*^n = 11.

### Postprandial Levels of IL-6

IL-6 concentrations, as reported previously (20), were low at baseline in both groups (Table 1) and increased during the postprandial phase in both groups (2.6-fold; 95% CI, 1.8-3.6; *P* < .001 and 1.9-fold; 95% CI, 1.3–2.95; *P* < .001 median increase at 360 minutes in the healthy weight and obesity group, respectively). Basal and postprandial IL-6 levels were higher in the group with obesity (Table 1). As anticipated (43, 44), IL-6R ab increased basal and postprandial IL-6 levels similarly in both groups (25-fold; 95% CI, 19-31, *P* < .001; and 22-fold; 95% CI, 15-27; *P* < .001) median increase at 30 minutes in the healthy weight and obesity group, respectively.

### Systemic Plasma Amino Acids

We first assessed whether IL-6R ab affected fasting and postprandial systemic plasma amino acids, which can influence protein synthesis.

**Figure 2.**
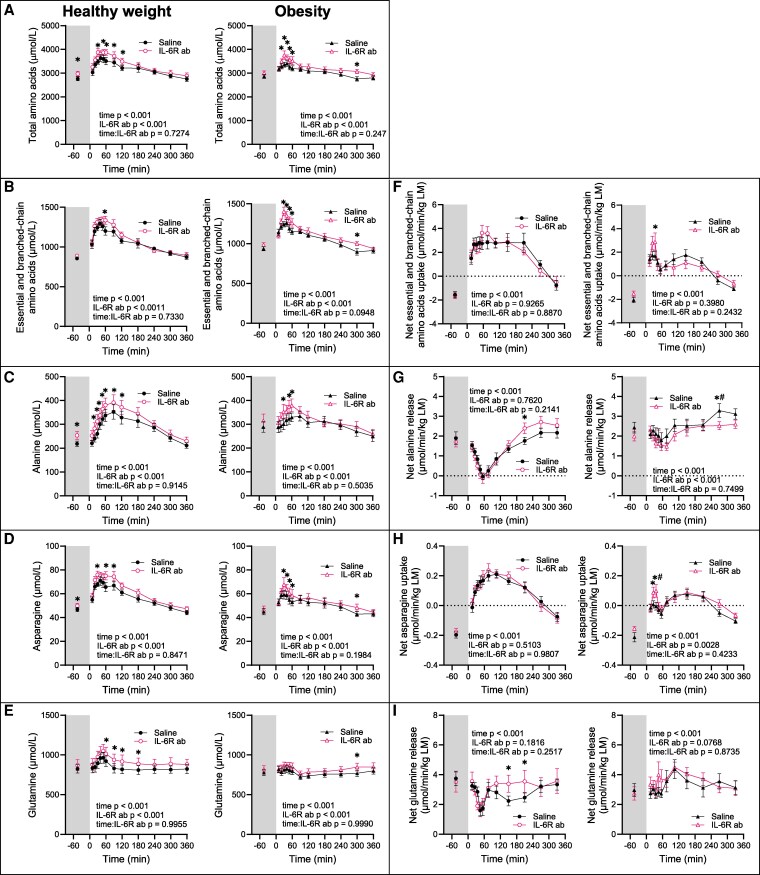
Plasma amino acid concentrations. (A-E) Arterial amino acids concentrations, (F-I) amino acid balances across the leg. Left panels show data from the group with healthy weight (n = 12); right panels show data from the group with obesity (n = 12). Data are represented as mean ± SEM. IL-6R ab, IL-6 receptor antibody; *IL-6R ab effect at specific timepoint, *P* < .05 using a linear mixed-effects model; #IL-6R ab effect different between groups, *P* < .05. See also Table 2.

Overall, on visit Saline, arterial concentrations of most amino acids (μmol/L) increased postprandially in both groups (Fig. 2A-2E, S2A-V (42)). Exceptions were glycine, cysteine, hydroxy-proline, methylhistidine, citrulline (Fig. S2I, S2P, S2Q, S2R and S2S (42)) in both groups, and aspartate and glutamate (Fig. S2J and S2K (42)) in the group with obesity.

Basal concentrations of essential amino acids (EAA) + branched-chain amino acids (BCAA), valine, glutamate, alanine, and tyrosine were higher in the group with obesity, whereas glycine and serine were lower compared to the healthy weight group (Fig. 2A-2E, Table 2). Postprandial increases in total amino acids (healthy weight Δ + 874; 95% CI, 523-1225; obesity Δ + 537; 95% CI, 254-819 at 35 minutes, *P* = .01 comparing groups) (Fig. 2A), EAA + BCAA (healthy weight Δ + 406; 95% CI, 265-547; obesity Δ + 252; 95% CI, 141-362 at 45 minutes, *P* = .012 comparing groups) (Fig. 2B), and several individual amino acids, such as asparagine (healthy weight Δ + 22.5; 95% CI, 13.8-31.2; obesity Δ + 9.9; 95% CI, 3.4-16.3 at 45 minutes, *P* < .001 comparing groups) (Fig. 2D), were greater in the healthy weight group Fig. S2 (42). However, AUC analyses showed no group difference for most amino acids, except for glycine and asparagine, which were lower in the group with obesity (Table 2).

**Table 2.** Baseline and AUC postprandial amino acid concentrations in plasma

	Healthy weight (n = 12)						Obesity (n = 12)					
	Fasting (μmol/L)			Postprandial (AUC)			Fasting (μmol/L)			Postprandial (AUC)		
	Saline	IL-6R ab	*P* value	Saline	IL-6R ab	*P* value	Saline	IL-6R ab	*P* value	Saline	IL-6R ab	*P* value
***BCAA***												
**Leu**	113 ± 5	117 ± 5	.2402	51 829 ± 1836	52 802 ± 1893	.4714	126 ± 5	131 ± 4	.2252	50 355 ± 1741	53 695 ± 1862	.0059
**Ile**	57 ± 3	66 ± 6	.1540	27 768 ± 1165	31 563 ± 3177	.1950	65 ± 4	68 ± 3	.2358	26 762 ± 1063	28 388 ± 1167	.0197
**Val**	216 ± 9	218 ± 10	.8666	91 695 ± 3530	91 651 ± 3910	.8723	248 ± 10*^a^*	261 ± 11	.1926	94 776 ± 3348	100 656 ± 3052	.0199
***Other EAA***												
**Phe**	50 ± 1	52 ± 1	.0587	22 779 ± 262	23 359 ± 528	.2673	55 ± 3	55 ± 2	.5104	23 515 ± 1261	24 258 ± 1117	.1083
**Met**	23.0 ± 0.6	24.1 ± .4	.2095	10 449 ± 370	11 257 ± 359	.0074	23.5 ± 1.2	24.1 ± .6	.3731	9470 ± 498	10 285 ± 469	.0273
**Lys**	154 ± 7	156 ± 7	.5724	69 555 ± 1992	70 922 ± 2231	.2140	165 ± 5	173 ± 4	.0721	71 036 ± 1692	74 088 ± 1909	.0347
**His**	76 ± 2	79 ± 5	.5654	29 431 ± 637	31 255 ± 1720	.2316	80 ± 3	89 ± 3	.0055	30 197 ± 1320	32 887 ± 1111	.0135
**Thr**	111 ± 4	117 ± 6	.1432	41 909 ± 1753	45 699 ± 2486	.0400	114 ± 7	120 ± 8	.2663	40 895 ± 2433	43 518 ± 2498	.0735
**Tryp**	57 ± 1	60 ± 2	.2390	20 502 ± 463	20 876 ± 727	.6177	62 ± 2	64 ± 3	.4021	20 976 ± 685	21 880 ± 897	.2126
***NEAA***												
**Gly**	229 ± 13	231 ± 14	.8043	74 266 ± 4335	75 287 ± 5004	.7266	182 ± 10*^a^*	179 ± 10	.5683	58 986 ± 3148*^a^*	57 693 ± 2919	.3625
**Asp**	4.5 ± 0.2	5.2 ± 0.9	.5954	2077 ± 146	1836 ± 105	.0010	6.4 ± 1.1	4.5 ± .3	.07396	2146 ± 198	1955 ± 158	.2083
**Asn**	46.8 ± 1.9	49.9 ± 1.9	.0102	19 756 ± 574	21 298 ± 718	.0076	44.9 ± 2.6	46.8 ± 2.8	.1152	17 611 ± 1019*^a^*	18 692 ± 1081	.0026
**Glu**	85 ± 3	80 ± 6	.2294	34 590 ± 2684	30 317 ± 1939	.0143	100 ± 6*^a^*	93 ± 6	.0622	36 606 ± 2211	36 557 ± 2099	.9898
**Gln**	825 ± 53	871 ± 69	.3369	292 408 ± 18 947	318 873 ± 25 606	.1821	779 ± 37	821 ± 42	.3528	268 440 ± 13 202	285 838 ± 14 185	.2325
**Arg**	82 ± 4	84 ± 4	.563	29 950 ± 1223	31 798 ± 1240	.0350	84 ± 4	87 ± 3	.0853	29 854 ± 1121	30 947 ± 1436	.2307
**Ala**	219 ± 11	253 ± 17	.0058	101 371 ± 6782	111 195 ± 7760	.0195	289 ± 23*^a^*	314 ± 28	.2367	104 516 ± 6668	110 467 ± 6647	.3013
**Ser**	119 ± 5	119 ± 6	.9021	42 753 ± 2124	44 174 ± 2469	.2590	103 ± 5*^a^*	101 ± 4	.6780	36 208 ± 1657*^a^*	36 877 ± 1145	.2802
**Tyr**	47 ± 2	49 ± 2	.2295	24 507 ± 1343	25 083 ± 1242	.2314	60 ± 2*^a^*	60 ± 3	.9317	25 998 ± 998	27 224 ± 1312	.1255
**Pro**	172 ± 14	188 ± 20	.0636	89 452 ± 6590	95 963 ± 7565	.0142	184 ± 12	201 ± 18	.2282	84 169 ± 4134	90 633 ± 5773	.1013
**Cys**	74 ± 6	151 ± 69	.1842	23 857 ± 2259*^b^*	25 416 ± 2147*^b^*	.4290	90 ± 10	106 ± 17	.0301	26 469 ± 3167	31 812 ± 3014	.2087
***NPAA***												
**HoPro**	12 ± 1	13 ± 2	.3476	3756 ± 387	4392 ± 477	.1437	16 ± 2	18 ± 2	.3109	4982 ± 584	5432 ± 610	.3737
**Tau-MeHis**	4.84 ± 0.32	5.06 ± 0.36	0.5311	1543 ± 111	1578 ± 100	.6675	4.98 ± 0.25	5.71 ± 0.37	.0428	1485 ± 86	1711 ± 113	.0277
**Citrulline**	45 ± 3	44 ± 4	.6388	130 241 ± 839	14 716 ± 1488	.2101	39 ± 4	39 ± 2	.8003	12 755 ± 1787	12 731 ± 958	.6822
**Ornithine**	64 ± 4	64 ± 4	.8944	26 463 ± 1322	25 535 ± 845	.6159	63 ± 5	58 ± 3	.2048	23 259 ± 1684	23 804 ± 1240	.4700
***Totals***												
**BCAA**	386 ± 16	401 ± 18	.2905	172 219 ± 6032	175 808 ± 7183	.4276	401 ± 18*^a^*	460 ± 17	.1918	171 734 ± 5719	182 477 ± 5849	.0098
**EAA**	470 ± 11	488 ± 11	.0795	193 928 ± 3923	201 667 ± 5545	.0615	499 ± 14	526 ± 13	.06558	194 384 ± 5533	205 112 ± 5583	.0232
**EAA + BCAA**	856 ± 20	888 ± 19	.0979	366 147 ± 7535	377 475 ± 8994	.0750	938 ± 26*^a^*	986 ± 25	.0843	366 118 ± 9765	387 590 ± 9718	.0073
**Total AA**	2763 ± 84	2974 ± 105	.0303	1 104 957 ± 35 057	1 161 824 ± 42 443	.0593	2864 ± 82	3005 ± 90	.0893	1 057 165 ± 30 140	1 124 415 ± 32 386	.0220

Abbreviations: BCAA, branched-chain amino acids; EAA, essential amino acids; NEAA, nonessential amino acids; NPAA, nonproteogenic amino acids.

^*a*^ *P* < .05 comparing healthy weight and obesity.

^*b*^n = 11.

**Figure 3.**
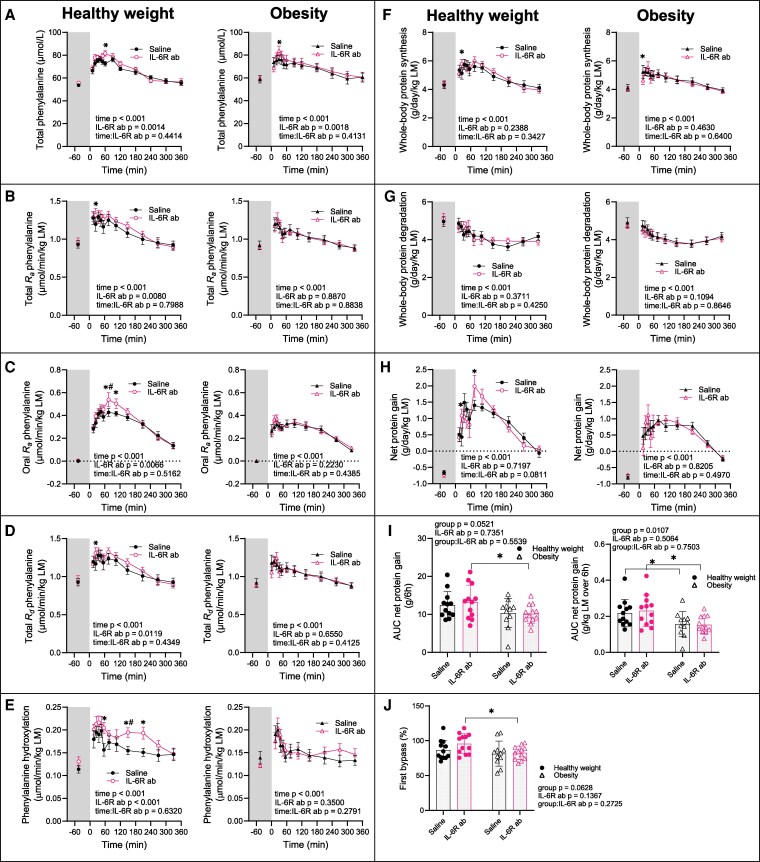
Protein kinetics at the whole-body level. Arterial phenylalanine concentrations (A), total *R_a_* phenylalanine (B), oral *R_a_* phenylalanine (C), total *R_d_* phenylalanine (D), phenylalanine hydroxylation (E), whole-body protein synthesis per day and kg lean mass (g/d/kg LM) (F), whole-body protein degradation (g/d/kg LM) (G), net protein gain (g/d/kg LM) (H), AUC net protein gain over the 6-hour postprandial phase in total (left panel) and per kg lean mass (right panel) (I), and first bypass of labelled phenylalanine from ingested casein (J). Left panels show data from the group with healthy weight (n = 12), right panels data from the group with obesity (n = 12). Data are represented as mean ± SEM. IL-6R ab, IL-6 receptor antibody; *IL-6R ab effect at specific timepoint, *P* < .05 using a linear mixed-effects model; #IL-6R ab effect different between groups, *P* < .05.

On the IL-6R ab visit, basal concentrations of total amino acids (Fig. 2A, Table 2), alanine (Fig. 2C, Table 2), and asparagine (Fig. 2D, Table 2) were elevated in the healthy weight group, with similar trends for EAA + BCAA (Fig. 2B, Table 2), EAA (Fig. S2V (42), Table 2), phenylalanine (Fig. 3A, Table 2), and proline (Fig. S2O (42), Table 2). In the group with obesity, basal levels of histidine, cysteine, and methyl-histidine were elevated (Table 2), with trends toward higher total amino acids (Fig. 2A, Table 2), EAA + BCAA (Fig. 2B, Table 2), EAA (Fig. S2V (42), Table 2), lysine (Fig. S2E (42), Table 2), and arginine (Fig. S2L (42), Table 2). In the healthy weight group, IL-6R ab increased postprandial AUCs for methionine, threonine, aspartate, asparagine, arginine, alanine, and proline, with similar trends for total amino acids, EAA + BCAA, EAA, and lysine, whereas AUC for glutamate decreased (Table 2). In the obesity group, IL-6R ab increased postprandial AUCs for total amino acids, EAA, total and individual BCAA, methionine, lysine, histidine, asparagine, and methyl-histidine (Table 2).

Overall, IL-6 receptor blockade increased fasting and postprandial concentrations of several amino acids in both groups. Thus, IL-6 may lower amino acid availability in circulation.

### Amino Acid Balance Across the Leg

We assessed the net amino acid balance across the leg in the fasted and postprandial states to evaluate changes in lean mass.

On the Saline visit, there was a net release of most amino acids (μmol/L/kg LM) from the leg in the fasting state (Fig. 2F-2I, Fig. S3 (42)), except for glutamate (Fig. S3K (42)), aspartate (Fig. S3J (42)), and serine (Fig. S3M (42)), which were consistently taken up in both groups. Postprandially, net release shifted to net uptake for most amino acids in both groups (Fig. 2F and 2H, S3 (42)). The healthy weight group exhibited a more pronounced postprandial positive amino acid balance across the leg, with a monophasic response, whereas the obesity group showed a biphasic response (Fig. 2F and 2H, S3 (42)). This is exemplified for EAA + BCAA (net uptake for healthy weight Δ + 4.3: 95% CI, 2.5-6.1; for obesity Δ + 2.6: 95% CI, 1.0-4.1 at 55 minutes, *P* = .032 comparing groups) and asparagine (net uptake for healthy weight Δ + 0.41: 95% CI, 0.29-0.52; for obesity Δ + 0.28: 95% CI, 0.18-0.39 at 105 minutes, *P* = .002 comparing groups) in Fig. 2F and 2H, respectively.

Alanine and glutamine did not shift to net uptake; their net release was suppressed postprandially in both groups, but markedly only in the healthy weight group (Fig. 2G and 2I). Alanine net release was completely suppressed a 55 minutes only in the healthy group (fasting: healthy weight 1.90; 95% CI, 1.43-2.37; obesity 2.43; 95% CI, 1.85-3.02, *P* = .17 comparing groups; at 55 minutes: healthy weight -0.37; 95% CI, -0.50 to 0.43; obesity 1.80; 95% CI, 1.21-2.38, *P* < .001 comparing groups) (Fig. 2G).

Contrary to our hypothesis, the IL-6R ab did not consistently affect net balance of all amino acids across the leg in either group (Fig. 2F-2I, S3 (42)). However, in the healthy weight group, postprandial net release of alanine and glutamine was higher following IL-6R ab (Fig. 2G and 2I), whereas in the obesity group, early postprandial net uptake of several amino acids was accentuated (Fig. 2F and 2H, S3ABU).

These findings suggest impaired postprandial uptake of amino acids across the leg in the group with obesity, with minimal effect of the IL-6R ab.

### Whole-body Phenylalanine Kinetics and Protein Turnover

We used 2 differently labeled phenylalanine tracers to determine amino acid kinetics and protein turnover systemically.

On the Saline visit, total plasma phenylalanine concentrations (μmol/L) were similar when fasting but increased less postprandially in the obesity group (healthy weight Δ + 38%: 95% CI, 23-55; obesity Δ + 22: 95% CI, 11-35, *P* = .020 comparing groups at 45 minutes, *P* = .089 for time:group interaction) (Fig. 3A).

Total *R_a_* of phenylalanine (μmol/min/kg LM) reflects phenylalanine appearance per kilogram of LM from both protein degradation and meal intake. It increased postprandially from 15 minutes to 105 minutes similarly in both groups (healthy weight maximal Δ + 39%: 95% CI, 20-61; obesity maximal Δ + 32: 95% CI, 16-50, *P* = .150 for group effect) (Fig. 3B).

Because casein in the meal was labeled with L-[ring-D_5_]phenylalanine, phenylalanine coming only from the meal (ie, oral *R_a_* phenylalanine) could be calculated. We observed greater oral *R_a_* phenylalanine (μmol/min/kg LM) in the group with healthy weight (healthy weight maximal 0.43: 95% CI, 0.36-0.50 at 45 minutes; obesity 0.32: 95% CI, 0.27-0.38 at 35 minutes, *P* = .008 and *P* = .05 comparing groups at 45 and 35 minutes, respectively, but *P* = .216 for group effect) (Fig. 3C).

Total *R_d_* phenylalanine (μmol/min/kg LM) reflects tissue uptake for both synthesis and oxidation. It increased postprandially until 150 minutes in both groups (healthy weight maximal Δ + 38%: 95% CI, 20-58 at 35 minutes; obesity maximal Δ + 31: 95% CI, 14-50 at time 25 minutes, *P* = .066 for time:group interaction) (Fig. 3D). When then calculated the rate of phenylalanine hydroxylation (μmol/min/kg LM), which represents hepatic amino acid oxidation as surrogate for amino acid oxidation. Phenylalanine hydroxylation was increased postprandially until 105 minutes in the healthy weight group and until 25 minutes in the obesity group (healthy weight maximal Δ + 75%: 95% CI, 35-125 at 45 minutes; obesity Δ + 48: 95% CI, 16-88 at 25 minutes, *P* = .162 for time:group interaction) (Fig. 3E).

Using these parameters, we calculated whole-body protein synthesis rate, degradation rate, and net protein gain (g/day/kg LM) (see Methods) (Fig. 3F-3H). As expected, fasting was associated with net protein loss (Fig. 3H), whereas meal ingestion increased synthesis (Fig. 3F), suppressed degradation (Fig. 3G), and resulted in net gain until 270 minutes (Fig. 3H). Basal and postprandial synthesis and degradation rates did not differ between groups (Fig. 3F-3G), but net protein gain was lower in the group with obesity (healthy weight maximal Δ + 2.15: 95% CI, 1.40-2.89 at 35 minutes; obesity Δ + 1.74: 95% CI, 1.05-2.43 at 75 minutes, group effect *P* = .037) (Fig. 3H), as was the AUC of net protein gain (g/kg LM over 6 hours) (healthy weight .218: 95% CI, 0.160-0.268; obesity 0.157: 95% CI, 0.119-0.196, *P* = .011 for group effect) (Fig. 3I). First-pass extraction of meal-derived phenylalanine was similar between groups (healthy weight 87%: 95% CI, 76-95; obesity 82%: 95% CI, 73-87, *P* = .063 for group effect) (Fig. 3J).

On IL-6R ab, postprandial phenylalanine concentrations (μmol/L) increased more than on Saline in both groups similarly (healthy weight Δ + 12%: 95% CI, 4-22 at 55 minutes, *P* = .005; obesity Δ + 10: 95% CI, 3-18 at 25 minutes, *P* = .009; *P* = .866 for group:IL-6R ab interaction) (Fig. 3A). Fasting total *R_a_* phenylalanine (μmol/min/kg LM) remained unchanged, whereas postprandial total *R_a_* increased only in the healthy weight group (healthy weight maximal Δ + 11%: 95% CI, 0-23 at 55 minutes, *P* = .045; obesity *P* = .887 for IL-6R ab effect; *P* = .052 for group:IL-6R ab interaction) (Fig. 3B). Oral *R_a_* phenylalanine (μmol/min/kg LM) increased on IL-6R ab only in the healthy weight group (healthy weight Δ + 0.11: 95% CI, 0.03-0.19 at 105 minutes, *P* = .008; obesity *P* = .223 for IL-6R ab effect; *P* = .121 for group:IL-6R ab interaction) (Fig. 3C). Basal *R_d_* phenylalanine (μmol/min/kg LM) was unaffected by IL-6R ab but postprandial *R_d_* phenylalanine increased in the healthy weight group (healthy weight Δ + 13%: 95% CI, 3-25 at 25 minutes, *P* = .013; obesity *P* = .655 for IL-6R ab effect; *P* = .032 for group:IL-6R ab interaction, Fig. 3C). Phenylalanine hydroxylation rate was unaffected by IL-6R ab during fasting but increased early (healthy weight Δ + 34%: 95% CI, 12-60 at 75 minutes, *P* = .002) and late (healthy weight Δ + 27%: 95% CI, 6-52 at 210 minutes, *P* = .009) postprandially only in the healthy weight group (obesity *P* = .35 for IL-6R ab effect; *P* < .001 for group:IL-6R ab interaction) (Fig. 3E). IL-6R ab had no effect on whole-body protein synthesis, degradation, net protein gain (Fig. 3F-3I), or first-pass hepatic extraction (Fig. 3J).

### Leg Protein Turnover

We quantified muscle protein turnover in the leg by measuring the incorporation of infused labeled phenylalanine.

**Figure 4.**
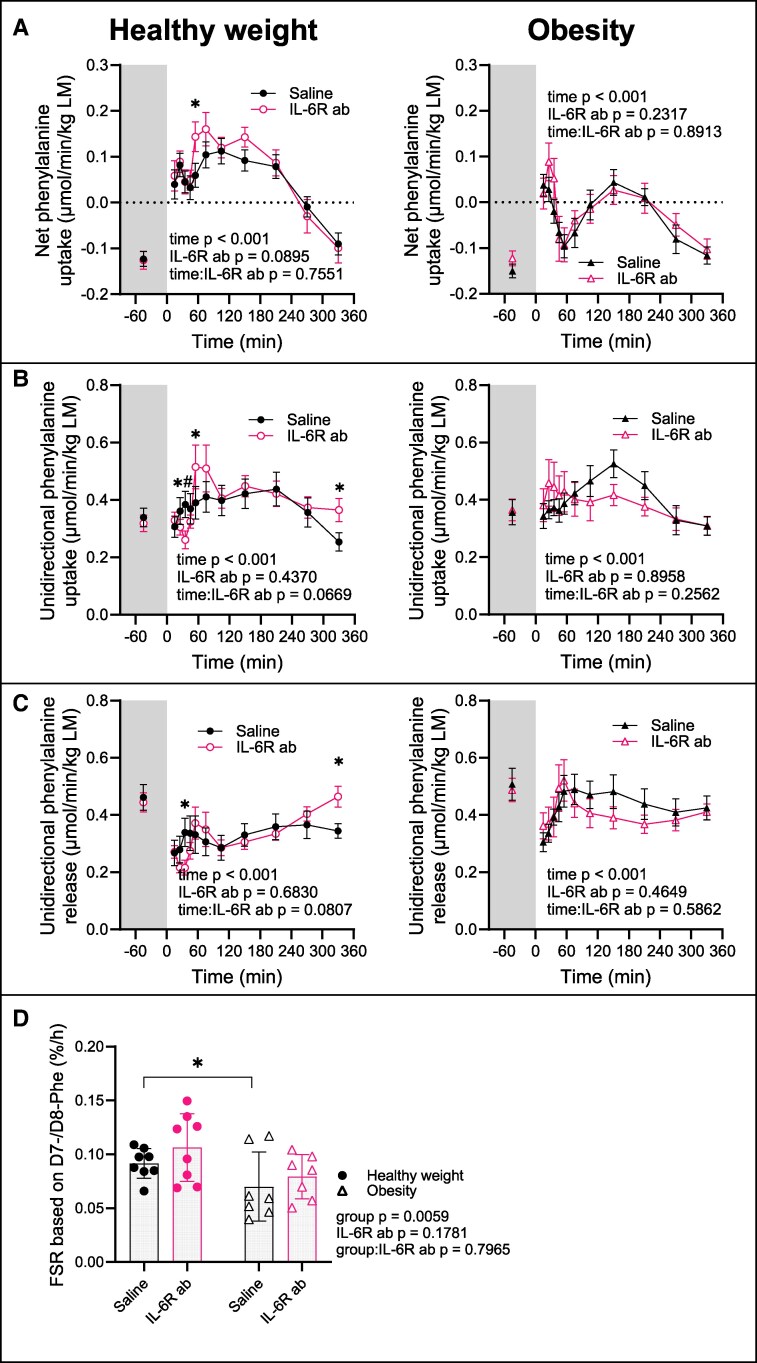
Protein turnover at the level of the leg. Net phenylalanine uptake representing net protein synthesis (A), phenylalanine uptake representing protein synthesis (B), phenylalanine release representing protein degradation (C), and muscle fractional synthesis rate, FSR (D). (A-C) Left panels show data from the group with healthy weight (n = 12), right panels data from the group with obesity (n = 12). (D) Both groups are included in 1 panel (n = 8 each). Data are represented as mean ± SEM. IL-6R ab, IL-6 receptor antibody; *P < .05 using a linear mixed-effects model for IL-6R ab effect at specific timepoint (A-C) and contrast between the group with healthy weight and the group with obesity (D).

On the Saline visit, in both groups, net phenylalanine uptake in the leg (μmol/min/kg LM) was negative in the fasting state (healthy weight −0.123: 95% CI, -0.176 to -0.070; obesity -0.150: 95% CI, -0.209 to -0.092; *P* = .233 comparing groups) (Fig. 4A), indicating net skeletal muscle protein loss. This was driven by lower phenylalanine uptake (μmol/min/kg LM) (healthy weight 0.339: 95% CI, 0.246-0.432; obesity 0.357: 95% CI, 0.255-0.459; *P* = .743 comparing groups) (Fig. 4B) compared to phenylalanine release (μmol/min/kg LM) (healthy weight 0.462: 95% CI, 0.379-0.545; obesity 0.508: 95% CI, 0.407-0.608; *P* = .531 comparing groups) (Fig. 4C). Following meal ingestion, net uptake turned positive in the healthy weight group and remained so until 270 minutes (healthy weight maximal Δ + 0.235: 95% CI, 0.133-0.338 at 105 minutes, *P* < .001; obesity maximal Δ + 0.194: 95% CI, 0.078-0.310 at 150 minutes, *P* = .001), primarily because of suppressed phenylalanine release, reflecting reduced protein degradation. This postprandial reduction in degradation was observed between 15 and 105 minutes in the healthy weight group but was nearly absent in the group with obesity (only from 15 minutes to 25 minutes) (Fig. 4A).

The phenylalanine uptake curve paralleled that of the EAA + BCAA (Fig. 2F), suggesting broader amino acids involvement in the impaired net balance in obesity. Consistent with this, the FSR (%/h) of mixed-muscle protein was lower in the group with obesity compared to the healthy weight group (healthy weight 0.094: 95% CI, 0.085-0.108; obesity 0.070: 95% CI, 0.49-0.088; *P* = .006 for group effect) (Fig. 4D), indicating reduced muscle protein renewal postmeal. The IL-6R ab had no relevant effect on muscle protein turnover in the leg.

### Turnover of Plasma Proteins

The FSR of total plasma proteins (%/day) quantifies the proportion of the total plasma protein pool synthesized over a given time. Because albumin is the predominant plasma protein (45), changes in the total plasma protein FSR likely reflects mainly alterations in albumin synthesis.

**Figure 5.**
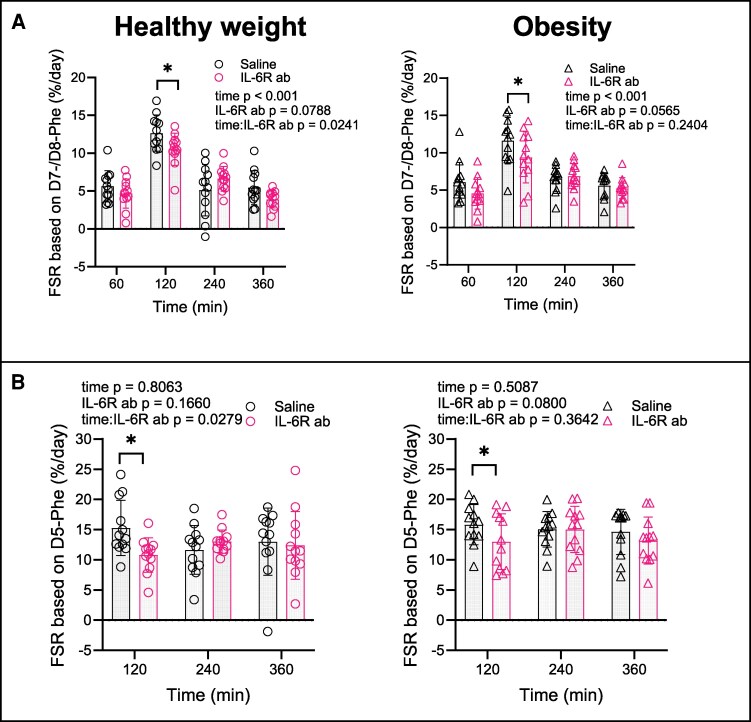
Fractional synthesis rate (FSR) of plasma proteins. FSR based on infused D7-/D8-phenylalanine (A) and FSR based on meal-derived D5-phenylalanine (B). Left panels show data from the group with healthy weight (n = 12), right panels data from the group with obesity (n = 12). Data are represented as mean ± SEM. IL-6R ab, IL-6 receptor antibody; *IL-6R ab effect at specific timepoint, *P* < .05 using a linear mixed-effects model.

On Saline, FSR increased similarly in response to the meal in both groups (healthy weight maximal FSR 12.9: 95% CI, 11.6-14.2; obesity 11.7: 95% CI, 10.3-13.0 at 120 minutes, *P* = .804 for group effect) (Fig. 5A).

On IL-6R ab, the FSR was reduced in both groups (healthy weight Δ −2.1: 95% CI -3.9 to -0.4 at 120 minutes, *P* = .019; obesity Δ −2.2: 95% CI, -4.1 to -0.3 at 120 minutes, *P* = .024; *P* = .807 for group:IL-6R ab interaction) (Fig. 5A). This decrease in FSR was confirmed when FSR was calculated based on meal-derived labeled phenylalanine (Fig. 5B). Given that the liver receives approximately 75% of its blood supply from the portal vein and 25% from the hepatic artery, orally administered D_5_-phenylalanine contributes more directly to hepatic protein synthesis, resulting in a higher FSR compared to that based on infused phenylalanine. Overall, these findings suggest that basal IL-6 activity supports plasma proteins synthesis in the liver.

### Amino Acid Balance Across Adipose Tissue

Although arteriovenous differences for selected amino acids (alanine, glutamine, glutamate) have previously been measured across human adipose tissue in both fasting and postprandial states (46), and comprehensive fasting balances have been studied in individuals with healthy weight and obesity (47), postprandial amino acid dynamics in human subcutaneous adipose tissue remain poorly characterized. Fig. S4 (42) shows the arteriovenous differences for each amino acid across abdominal subcutaneous adipose tissue. Due to the low number of participants with successful catheterization and exploratory nature of our analyses, we decided to show the raw means ± SEM for each amino acid and formal statistics for only a few selected amino acids (healthy weight n = 6 and n = 3 on Saline and IL-6R ab, respectively; obesity n = 8 for both Saline and IL-6R ab).

In the fasting state, net balance (μmol/L) was near zero for most amino acids, except for net release of glycine (healthy weight 17: 95% CI, 7-26; obesity 19: 95% CI, 11-27, *P* = .748 comparing groups), glutamine (healthy weight 47: 95% CI, 6-87; obesity 53: 95% CI, 18-89, *P* = .772 comparing groups), and alanine (healthy weight 39: 95% CI, 8-70; obesity 80: 95% CI, 53-106, *P* = .056 comparing groups), and net uptake of glutamate (healthy weight 61: 95% CI, 41-80; obesity 62: 95% CI, 46-79, *P* = .740 comparing groups).

Following the meal, most amino acids exhibited a transient peak in net uptake (eg, leucine, isoleucine, valine, asparagine). BCAA uptake remained positive throughout the postprandial period and was numerically lower in the obesity group (*P* = .252 for group effect, *P* = .187 for time:group interaction). Glutamate showed consistent net uptake in both fasting and postprandial states. Meal ingestion temporarily suppressed alanine release, with less pronounced suppression in the group with obesity (healthy weight 3: 95% CI, -28 to 34; obesity 57: 95% CI, 31-84 at 75 minutes, *P* = .012 comparing groups).

Overall, IL-6R ab did not affect amino acid balance (Fig. S5 (42)), except for reduced glutamate uptake in the healthy weight group (*P* < .001 for IL-6R ab effect, n = 3) (Fig. S5M).

### Postprandial Hormonal Responses

Basal hormone concentrations, partly reported previously (20), are summarized in Table 1 and Table S1 (42). The group with obesity was less insulin sensitive (Matsuda index healthy weight 5.5 ± 0.5, obesity 2.8 ± 0.5, *P* < .001; HOMA-IR healthy weight 1.3 ± 0.2, obesity 3.9 ± 1.6, *P* < .001), and had higher basal (healthy weight 32 ± 3 pmol/L, obesity 90 ± 13 pmol/L, *P* < .001) and postprandial insulin concentrations (incremental AUC healthy weight 65 680 ± 9397, obesity 114 548 ± 19 876, *P* = .048).

Basal (healthy weight 4.1 ± 0.6 pmol/L, obesity 6.0 ± 0.6 pmol/L, *P* = .026) and postprandial (total AUC healthy weight 2085 ± 150, obesity 2971 ± 176, *P* = <.001) glucagon concentrations were also higher in the group with obesity. GH was suppressed at 120 minutes in the group with healthy weight (fasting healthy weight median 0.40 μg/L: 95% CI, 0.08-0.96; obesity 0.11 μg/L: 95% CI, 0.07-0.42, *P* = .164 comparing groups; at 120 minutes healthy weight Δ −83%: 95% CI, -37 to -95, *P* = .002; obesity Δ + 37%: 95% CI, -76 to +679%, *P* = .986) and increased at 300 to 360 minutes in both groups (at 360 minutes, healthy weight 9.2-fold: 95% CI, 2.9-29.6, *P* < .001; obesity 9.6-fold: 95% CI, 2.8-33.6, *P* < .001; *P* = .056 for time:group interaction). On IL-6R ab, basal insulin concentrations (pmol/L) was increased in the healthy weight group (Saline 31.6 ± 2.9, IL-6R ab 37.7 ± 3.6, *P* = .018), and basal glucagon (pmol/L) concentrations tended to be increased in the group with obesity (Saline 6.0 ± 0.6, IL-6R ab 6.7 ± 0.5, *P* = .069). IL-6R ab had no effect on basal or postprandial GH levels in either group.

## Discussion

This study reports prespecified exploratory outcomes from a trial originally designed for a different primary endpoint (IL-6's role in postprandial fat metabolism) (20). Findings should be interpreted as hypothesis-generating and require confirmation in independent studies.

This study investigated the impact of obesity and IL-6 receptor blockade on amino acid and protein turnover at the whole-body, skeletal muscle, and adipose tissue levels following a mixed meal. Key findings were: (1) altered fasting amino acid concentrations in obesity; (2) a blunted postprandial increase of plasma amino acids in obesity; (3) diminished whole-body and skeletal muscle protein gain in obesity; (4) increased fasting and postprandial amino acid concentrations after IL-6R blockade in both groups; (5) reduced plasma protein synthesis following IL-6R blockade in both groups; and (6) increased meal-derived phenylalanine appearance and phenylalanine oxidation in the healthy weight group.

### Fasting and Postprandial Amino Acid Concentrations are Altered in Obesity

We saw higher plasma concentrations of total BCAA, alanine, glutamate, and tyrosine, but lower glycine and serine in the obesity group.

Especially elevated fasting BCAA levels is a known phenomenon in obesity (48-50). Our data showing lower postprandial uptake of the BCAA, leucine, in adipose tissue and most amino acids in the leg are in line with previous rodent and ex vivo studies showing that elevated fasting BCAA may, at least partly, reflect reduced uptake and catabolism of BCAA in adipose tissue (51-53) and skeletal muscle (54, 55). Since concentrations of BCAA correlate with insulin concentrations (49), insulin resistance likely plays a role in altered BCAA metabolism in obesity. Conversely, elevated BCAA may also promote skeletal muscle insulin resistance (55, 56). Additionally, insulin resistance is likely the main cause of impaired suppression of protein breakdown and amino acid release from skeletal muscle (57-60), which in turn may contribute to elevated plasma concentrations of BCAA. Elevated BCAA a suggested to have deleterious effects on adipocyte differentiation and fat storage and contribute to adipose tissue inflammation (55).

Higher plasma alanine can be indicative of higher nonoxidative glucose metabolism in obesity through enhanced glycolytic flux with increased conversion of pyruvate to alanine. Although skeletal muscle is the main net producer of alanine (61, 62), Stumvoll et al found indirect indications that tissues other than skeletal muscle contribute to the higher plasma alanine levels in insulin-resistant states (63, 64). Given the higher net alanine release we saw not only from skeletal muscle but also from adipose tissue in the obesity group, adipose tissue likely contributes to this phenomenon.

IL-6 from chronic inflammation does not seem to mediate these effects, as we saw no reversal of these changes after blocking IL-6 signaling.

### Meal-induced Whole-body and Muscle Protein Gain are Reduced in Obesity

Despite no difference in baseline muscle protein turnover, postprandial protein gain normalized to lean mass at both whole-body and muscle levels, and muscle protein FSR were lower in the obesity group. This aligns with previous findings of either similar (65, 66) or reduced (67, 68) myofibrillar protein FSR in obesity. Muscle protein FSR is associated with muscle strength and function (69, 70); therefore, altered FSR may explain the lower muscle quality in people with obesity (71).

Less efficient suppression of muscle protein degradation may explain this impairment. Our data showing that the obesity group had less suppression of phenylalanine release in the postprandial state are in line with this. As mentioned, insulin resistance likely plays a major role (58-60), whereas IL-6 does not cause the obesity-related changes in net protein gain and muscle protein synthesis.

Our participants ingested 26 g of protein independent of body weight, and one could argue that the slightly lower relative protein intake in the obesity group may partly explain the differences between groups. However, both groups consumed >0.25 g/kg lean body mass protein and the literature indicates that beyond protein consumption this threshold or 20 to 30 g protein per meal does not lead to further enhanced muscle protein FSR (72, 73). Furthermore, similar plasma EAA + BCAA AUCs argue against limited amino acid availability as the cause.

### Loss of IL-6 Signaling Increases Plasma Amino Acid Concentrations but Blunts Meal-induced Plasma Protein Synthesis

IL-6R blockade increased plasma amino acid concentrations, especially postprandially, confirming and extending previous fasting findings (16). This is consistent with IL-6 infusion studies showing reduced plasma amino acids (8), which was likely due to increased amino acid uptake by extramuscular tissues. Our arteriovenous balance data across the leg and adipose tissue showed no consistent effect of IL-6R blockade across all elevated amino acids, which would explain the increased plasma amino acid concentrations, except that the postprandial release of alanine and glutamine from the leg was increased in the healthy weight group. This suggests that IL-6R blockade affects amino acid handling outside muscle and adipose tissue, potentially implicating the liver or immune cells.

Alanine and glutamine are key nitrogen carriers (74-78). Given that they contribute largely to hepatic gluconeogenesis and ureagenesis (61), and that IL-6 stimulates their uptake in hepatocytes (79), reduced hepatic amino acid utilization may explain the increase in alanine and glutamine levels. Although endogenous glucose production was unchanged (20), we cannot infer whether gluconeogenesis from amino acids was affected by IL-6R ab. Ureagenesis was not determined either. Furthermore, reduced IL-6 activity may lower immune cell amino acid consumption (80, 81), raising circulating amino acid levels. Taken together, further studies specifically designed to address these hypothesis are required.

### Loss of IL-6 Signaling Increases Phenylalanine Appearance From Meal and Phenylalanine Catabolism

IL-6R blockade also increased meal-derived phenylalanine appearance, suggesting reduced splanchnic amino acid uptake. Despite elevated amino acids, plasma protein synthesis declined, suggesting impaired hepatic protein synthesis, a mechanism thought to conserve amino acids postprandially for later use (82).

Skeletal muscle accounts for ∼50% of the postprandial rise in whole-body protein synthesis, with liver albumin synthesis contributing ∼30% (82, 83). Thus, the lack of an IL-6R blockade effect on whole-body protein synthesis likely reflects the predominant contribution of muscle while the effect on albumin synthesis may be masked.

Phenylalanine oxidation, which primarily occurs in the liver and reflects amino acid catabolism, increased only in the healthy weight group on IL-6R ab. Amino acid transport in the liver relies on facilitated diffusion, allowing hepatic amino acid concentrations to closely mirror those in plasma. As a result, phenylalanine oxidation rises proportionally with postprandial increases in plasma amino acid concentrations (61). Because the observed increase in phenylalanine oxidation occurred after the peak in phenylalanine appearance, this increased oxidation is likely a response to excess phenylalanine availability in circulation.

It has previously been shown that protein intake beyond a certain threshold does not further stimulate skeletal muscle protein synthesis but instead stimulates amino acid oxidation and ureagenesis (84, 85). Thus, basal IL-6 may enhance the storage of meal-derived amino acids in albumin to prevent catabolism of excess amino acids that are not stored elsewhere in the immediate postprandial phase.

### Limitations

We applied the 2-pool model to assess protein synthesis and degradation in skeletal muscle. The 2-pool model may underestimate muscle protein degradation. Tracer dilution during digestion and in the splanchnic bed may underestimate oral *R_a_* phenylalanine (86), although relative comparisons remain valid. The group with obesity had low systemic inflammation, and inclusion of metabolically less healthy individuals might have revealed stronger effects. Results may not generalize to women.

## Conclusion

In summary, these exploratory analyses suggest that basal IL-6 activity influences amino acid and protein metabolism but has no effect on muscle protein turnover during fasting and until 6 hours after a mixed meal. Instead, IL-6 potentially affects protein metabolism in other tissues like the liver and immune cells.

Although the observed effect sizes and CIs provide insight into the magnitude and direction of IL-6's effects, the findings are based on a limited sample and were not adjusted for multiple testing. They should therefore be interpreted as hypothesis-generating and confirmed in future studies specifically designed and powered to address these questions. Such work could help clarify the mechanistic role of IL-6 in human protein metabolism and its relevance for clinical interventions targeting IL-6 in various contexts, such as rheumatoid arthritis (87) or cardiovascular disease (88).

## Data Availability

Original data generated and analyzed during this study are included in this published article or in the data repositories listed in References.

## Abbreviations

AUC: area under the curve
BCAA: branched-chain amino acids
BMI: body mass index
EAA: essential amino acids
FSR: fractional synthesis rate
HOMA-IR: homeostasis model assessment-insulin resistance
IL-6R: IL-6 receptor
LM: lean mass
SEM: standard error of the mean
TTR: L-[ring-3,5-D_2_]tyrosine
